# Bud development, flower phenology and life history of holoparasitic *Rafflesia cantleyi*

**DOI:** 10.1007/s10265-024-01522-7

**Published:** 2024-02-14

**Authors:** Suk Ling Wee, Shwu Bing Tan, Sue Han Tan, Bernard Kok Bang Lee

**Affiliations:** 1 Department of Biological Sciences and Biotechnology, Faculty of Science and Technology, Universiti Kebangsaan Malaysia, 43600 Bangi, Selangor Darul Ehsan Malaysia; 2 Department of Mathematical Sciences, Faculty of Science and Technology, Universiti Kebangsaan Malaysia, 43600 Bangi, Selangor Malaysia

**Keywords:** Bud growth, Flower phenology, Life history, Holoparasitic plants, Rafflesiaceae, Sex ratio

## Abstract

Despite being the world's largest single-flower, *Rafflesia*’s biology and life history are still poorly understood due to its cryptic growth strategy on *Tetrastigma* vines. Previous studies have been mostly short-term, contrary to *Rafflesia*’s long development period before blooming. Bud development and flower phenology of *R. cantleyi* was studied in a dipterocarp forest in Lata Jarum, Peninsular Malaysia. Seven populations, consisting of 247 buds, were monitored fortnightly for 65 months in two discrete studies between 2009 and 2018. The bud size distribution of *R. cantleyi* is dynamic, progressively changing from small flower buds to larger buds before flowering. Buds < 5.0 cm across had the slowest growth and highest mortality rates, while those > 15.0 cm across demonstrated accelerated growth. The bud growth profiles of the same site clustered distinctively regardless of sex with successful blooming rate that varied greatly between sites, prompting speculation about their relatedness to the sites’ physical attributes. We reported the first female-dominated population in *Rafflesia*’s life history. *Rafflesia cantleyi*’s blooming rate at Lata Jarum is moderate to high, with non-seasonal flowering phenology as evident by the lack of synchronisation and consistency between flowering and local rainfall patterns. Based on the field data of the present study and the published information of other *Rafflesia* species, *R. cantleyi’s* life cycle was estimated between 4.0 and 5.3 years. Our findings further explain *Rafflesia*’s biology and life history and highlight the gap in knowledge of the natural habitats on the endoparasite’s growth and fate potentially for future conservation and study.

## Introduction

*Rafflesia* R.Br. ex Thomson bis (Rafflesiaceae) represents the genus of the world’s largest single-flower, and it is the most famous endoparasite clade among all endophytic parasites. The plant species is an obligatory endoparasite to vines of *Tetrastigma* Planch (Vitaceae), obtaining most, if not all, of its nutritional requirements via uniseriate or multiseriate endophytic filaments that are embedded into the host tissue (Meijer 1997; Nikolov et al. 2014a, 2014b; Wicaksono et al. 2021). The parasitic *Rafflesia* are rare, and their geographical distribution is confined to tropical rainforests in Indonesia, Malaysia, southern Thailand and the Philippines (Nais 2001).

The conservation of threatened or vulnerable plant and animal species requires considerable knowledge and understanding of their biology and ecology. For example, life history information is crucial for understanding and identifying potential threats to species growth, survival and reproduction. Due to the cryptic growth strategy of *Rafflesia*, much of the basic biology and life history of this taxa still needs to be explored and fully understood. Ecological research on *Rafflesia* has been challenging due to the long bud development period, ephemeral flowers and rarity of flowering events. Moreover, the remoteness of sites and the lack of financial resources to sustain long-term ecological monitoring and research work significantly impact the types of research that could be conducted and how progress could be monitored. To date, only a handful of studies have documented the life history and population dynamics of *Rafflesia*. However, most observations were conducted within a relatively short period of one to six months (Hidayati et al. 2000; Lestari et al. 2014; Susatya et al. 2017; Susatya 2020) except for Nais in 2001 and more recently, Barkman et al. (2017) and Tolod et al. (2021). Considering the long developmental period and the fact that the complete life cycle of *Rafflesia* takes years, these short studies only manage glimpse of the cryptic growth of *Rafflesia* spp.

Despite the fact that few ecological studies have been conducted, the generality has been that the bud size distribution of *Rafflesia* spp. is dominated by small flower buds (< 6 cm in diameter; Lestari et al. 2014; Nais 2001; Susatya et al. 2017; Susatya 2020). Bud size distribution may progressively change over time as buds grow and bloom (Susatya 2020). *Rafflesia* bud growth is the slowest during its post-emergence stage (2–4 cm), moderate as the buds are in mid-development (ca. 13 cm) and rapid in the pre-bloom stage, as observed in *R. arnoldii* Robert Brown, *R. keithii* Meijer, *R. pricei* Meijer and *R. tengku-adlinii* Mat-Salleh & Latiff (Meijer 1958; Nais 2001). An exponential growth pattern was first reported for *R. patma* Blume (Hidayati et al. 2000) and later for *R. arnoldii* (Susatya 2020) and *R. consueloae* Galindon, Ong & Fernando (Tolod et al. 2021). Susatya (2020) suggested that an exponential growth model explicit for *R. arnoldii* could be used to estimate the age of a flower bud based on its diameter measurement. However, it was unknown whether each *Rafflesia* species indeed followed a species-specific exponential growth model to enable such an estimation.

As more *Rafflesia* species are studied with respect to floral bud growth and fate, it is becoming clear that while the generality that high mortality occurs at the young bud stage holds true, the rate at which this occurs may vary greatly among *Rafflesia* species in their respective natural habitats, such as *R. arnoldii* (47.8%), *R. bengkuluensis* Susatya, Arianto & Mat-Salleh (67–100%), *R. consueloae* (77.3%), *R. keithii* (65%), *R. patma* (75%), *R. pricei* (93%) and *R. tengku-adlinii* (61%) (Hidayati et al. 2000; Nais 2001; Susatya et al. 2017; Susatya 2020; Tolod et al. 2021). Such findings are an important justification for investigating mortality rates and the causes of different *Rafflesia* species at different localities for conservation purposes.

As a consequence of the high flower bud mortality rate, parasitic *Rafflesia* suffered low flowering success, as reported in *R. bengkuluensis* (7.9%), *R. consueloae* (19.7%) and *R. patma* (6.7–12.5%) (Hidayati et al. 2000; Susatya et al. 2017; Tolod et al. 2021). The sex ratio of the dioecious *Rafflesia* flowers within a population is often male-biased, as observed in *R. keithii* (2.75: 1), *R. pricei* (44.5:1), *R. tengku-adlinii* (4.5:1), and *R. manillana* Teschemacher (15:1) (Nais 2001; Yahya et al. 2010). It is also very rare to observe both male and female flowers of the same species in bloom at the same time and locality. In all the case studies above, there was no re-visitation to the same site.

Even fewer studies have been conducted on flowering phenology of the *Rafflesia* species. The *Rafflesia* species in the Borneo, notably *R. arnoldii* and *R. patma* in Indonesia (Mursidawati et al. 2014; Susatya 2007), and *R. keithii*, *R. pricei* and *R. tengku-adlinii* in Sabah (Nais 2001) has been reported to flower all year round, while *R. kerii* Meijer in Thailand and *R. consueloae* in the Philippines appeared to flower seasonally, coinciding with the hottest/coldest and driest time of the year (Meijer and Elliott 1990; Tolod et al. 2021). We expected that *R. cantleyi*, an endemic species found in the tropical forests in Peninsula Malaysia, which is without a distinctive hot and dry season, would also have a non-seasonal flowering pattern.

Given the background literature, this study aims to investigate the bud size distribution, bud development, flower phenology, and life history of *R. cantleyi*, an endemic *Rafflesia* species found in Peninsular Malaysia. Specifically, we determined the mortality and bud growth rates of *R. cantleyi* in relation to bud diameter size and whether *R. cantleyi* follows a species-specific exponential growth model. In addition, we also evaluated the successful blooming rate and sex ratio of *R. cantleyi* and whether the flowering phenology is non-seasonal at Lata Jarum, Raub, Pahang.

This paper presents research on the collated bud growth data and the fate of the endoparasite *R. cantleyi* over two discrete study periods from 2009 to 2018, separated by a 3 year gap. The 3 year gap was unintentional and resulted from a lack of continuous funding and support for *Rafflesia* research. Nevertheless, this 65 month monitoring period yielded much biological and ecological information on *R. cantleyi*, including new, previously unknown findings for the *Rafflesia* species. Based on the field data of the generative stage of *R. cantleyi* and extrapolation from published information on the vegetative stage of *Rafflesia* spp., we then estimated the duration of the life cycle for *R. cantleyi*.

## Materials and methods

### Studied species

**Fig. 1:**
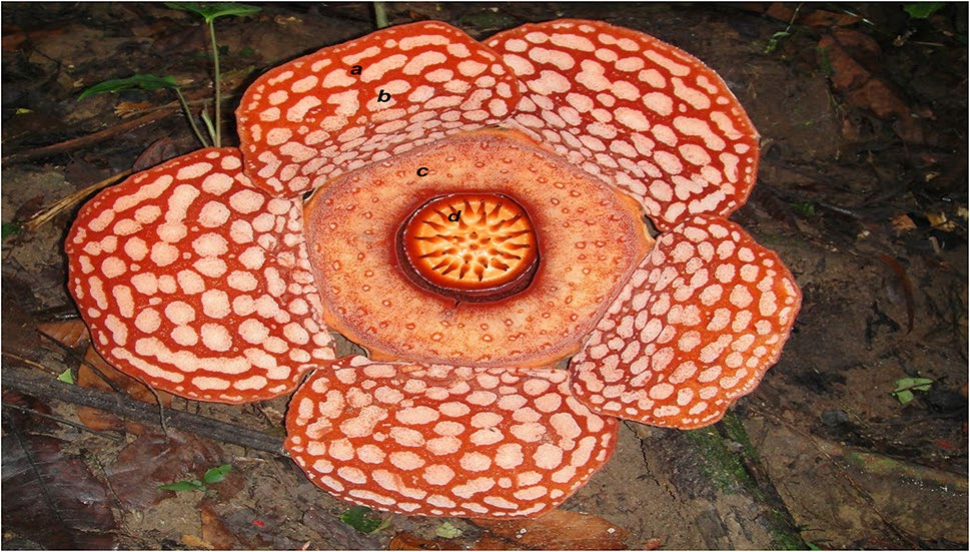
*Rafflesia cantleyi* Solms, an endemic species in Peninsular Malaysia. Large bright red perigone lobes (**a**) characterise the parasitic flower with six to eight whitish warts (**b**) in radial and lateral directions and about ten warts in the basal row. The diaphragm (**c**) opening is relatively small (4–8 cm across), with 30 processes (**d**) seen on the flower disc

*Rafflesia cantleyi* Solms was first described by H. Graft zu Solms-Laubach in 1910. It was named in honour of M. Cantley, a curator of the Singapore Botanic Gardens, who collected the type specimen in 1881 (Nais 2001). The five-perigone-lobe flower is gargantuan, with an average diameter between 30 and 55 cm at full bloom (Nais 2001). On some occasions, it can grow to as large as 70–80 cm in diameter (Wee SL, personal observation). The floral characteristics used for morphological identification include large bright red perigone lobes with six to eight whitish warts in radial and lateral directions and about ten warts in the basal row (Fig. 1). The diaphragm opening of *R. cantleyi* is between 4 and 8 cm across and comparable to that of *R. consueloae* (3.2–9 cm; Galindon et al. 2016), however, it is relatively small compared to other *Rafflesia* species (Nais 2001).

The distribution of *R. cantleyi* is limited to forests in Kedah, Perak, Kelantan, Terengganu and Pahang states within Peninsular Malaysia (Wong and Latiff 1994). The species thrives in primary and secondary lowland dipterocarp forests, with an altitudinal distribution between 470 and 610 m (Nais 2001).

### Study site

**Fig. 2:**
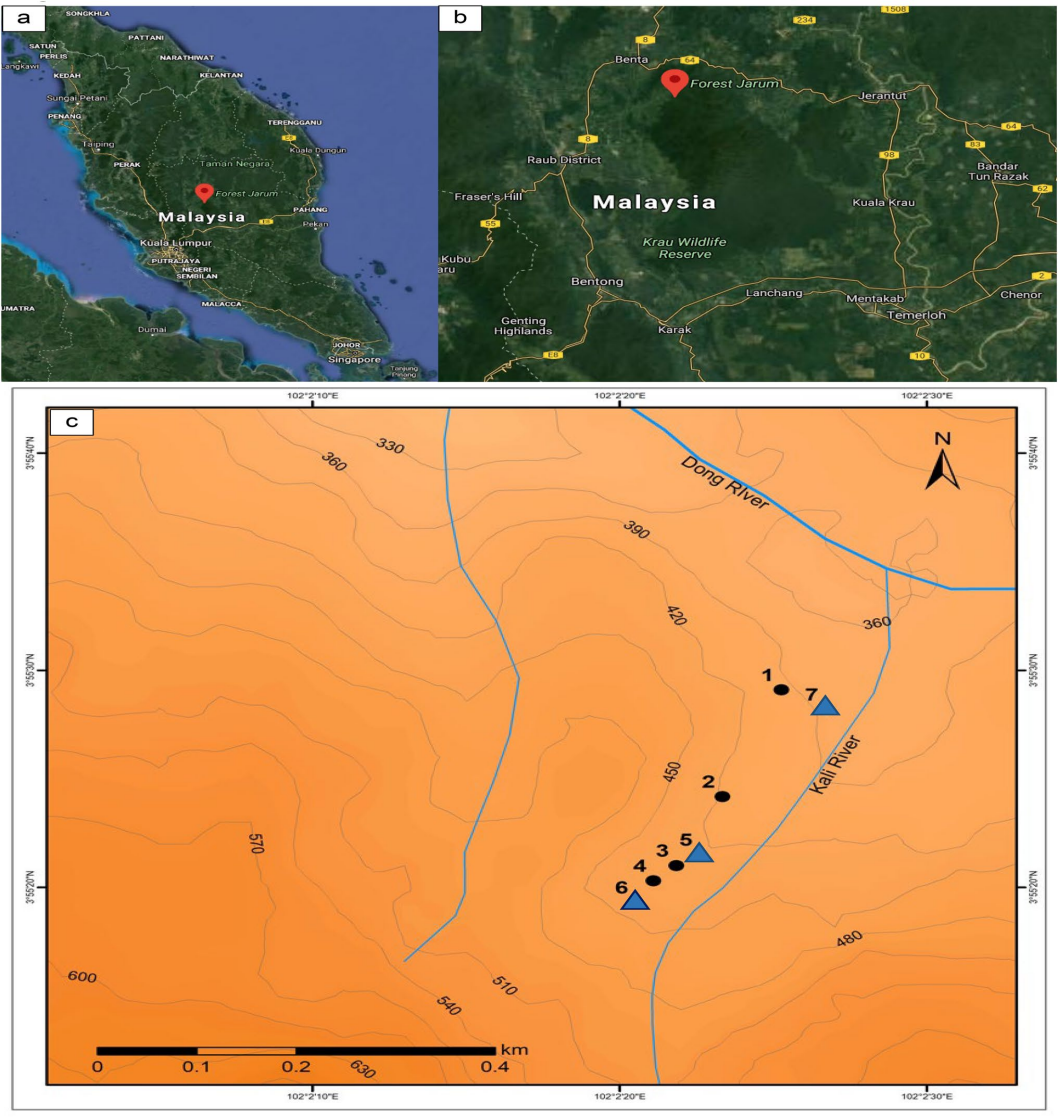
Maps depicting the Lata Jarum Recreational Forest nestled within the Benom Range, Raub, Pahang State, Peninsular Malaysia (indicated by location symbols in **a** and **b**); **c** shows the seven sampling sites in two different study periods (solid black circles = study period I, December 2009–February 2013; solid blue triangle = study period II, May 2016–June 2018)

The study was conducted at the Lata Jarum Recreation Forest within the Benom Forest Reserve at Raub, Pahang, Malaysia (03º 55.92ʹ N and 102º 01.99ʹ E) (Fig. 2). A waterfall approximately 50 m away from the recreational forest entrance is a local leisure spot. The forest entrance is gated for access control. Camping within the forest is not allowed, but accommodations, such as chalets and homestays, are available 2–10 km away. There is no man-made structure within the forest, and to reduce disturbance to the forest, the activities are limited to jungle trekking, environmental appreciation, and research and education purposes, for which an entry permit must be obtained from the Department of Forestry, Raub District Council.

Lata Jarum receives more rainfall from October to mid-January (average 233–285 mm per month) during the Northeast monsoon than during the Southwest monsoon (May to October; average 148–240 mm per month). There is no distinct dry season at Lata Jarum, but the period from March to May, which coincides with the transitions between the two monsoon periods, tends to be drier (Cheang 1993). Between 2009 and 2018, the site received an average annual rainfall of 2267 mm (min–max of 1800–2850 mm), with a mean air temperature and relative humidity of 27.0 °C and 83%, respectively (Malaysian Meteorological Service). The average monthly rainfall during the first and second studies was 181.0 mm and 221.2 mm, respectively (Malaysian Meteorological Service).

The study consisted of two discrete periods: study period I, from December 2009 to February 2013 (39 months except for Site 1), and study period II, from May 2016 to June 2018 (26 months), with four and three sites monitored, respectively. A sizable old tree fell at Site 1, destroyed the surrounding natural habitat and blocked the trail leading to the site. This resulted in a shorter study period for this population (18 months; 30 Dec 2009 to 19 May 2011). Subsequent surveillance activity in early 2016 showed no sight of any *Rafflesia* bud, which suggested that the *Tetrastigma* liana host population in Site 1 was likely destroyed.

A topographical map illustrating all sampling locations at the study sites is given in Fig. 2. The physical attributes of the sites are summarized in Table 1. The four sites in study period I were in the sequence of Site 1–Site 2–Site 3–Site 4 and were separated between 31 and 162 m apart, while the three sites in study period II were in the sequence of Site 7–Site 5–Site 6, separated between 52 and 241 m apart.

**Table 1:** Annotation on physical attributes of studied sites of *Rafflesia cantleyi* in Lata Jarum, Raub, Pahang, West Malaysia

Site	Estimated plot size (m^2^)	Site characteristics			
		Slope and other remarks	Canopy cover	Distance from water body	Soil
Study I (December 2009–February 2013**)**					
1	5 × 5	On top of a slope with an approximate inclination 45–55^o^	With thick tree canopy that receives little sunlight penetration;	Quite a distance away from water body	Soil not moisten most of the time
2	5 × 8	On a slope with 40–45° inclination	Not sheltered by tree canopies and received plenty of direct sunlight	With less than 2 m away from a slow-flowing stream	Soil constantly moistened
3	3 × 10	Along a trail with 20–25° inclination; with large boulders in the surroundings	Received very little sunlight penetration,	7–10 m away from a fast-flowing stream	Soils always moisten and sometimes with puddles of water on the ground
4	3 × 10	Along a trail; very close to a stream	occasionally with sunlight penetrated the tree canopies	Right next to a stream	Soil always damped
Study II (May 2016–June 2018)					
5	3 × 6	A long a trail; with large boulders on both sides	sheltered from direct sunlight	About 10–15 m from a stream	Soil always damped
6	15 × 20	On a reasonably flat land with a mixture of large trees and shrubs	on flat land and shelter by tree canopies without direct sunlight penetration;;	10 m away from a stream	Soil was reasonably moistened most of the time
7	5 × 4	Near to a stream	Sheltered from direct sunlight	About 3–5 m away from a stream	Soil was reasonably moistened

Recent findings by Barkman et al. (2017) revealed that most *Tetrastigma* plant grow less than 100 m apart, with significant genetic subdivision among component populations. Their microsatellite genotyping on paired parasites and host samples revealed that two *R. cantleyi* sampling sites separated by 40 m were actually two distinct populations. In addition, these two populations contained more than one parasite individual present within the same host vine at one time and may spread up to 14 m within the vine stem or root.

In this study, no effort was made to trace to host vine individuals (as some host vines are deep beneath the soil) or to excavate any below-ground buds (to avoid causing injury to the host). There was also no effort to genotype each bud/flower or host vine to distinguish between individual parasites and host vines. Therefore, we defined a *Rafflesia* population as an entire *Rafflesia* with hosts found within a designated sampling site. Based on the site-to-site distance (Site 1– Site 2: 162 m, Site 2–Site 3: 108 m and Site 3–Site 4: 31 m), Sites 1, 2 and 3 were considered three different populations. Sites 3 and 4, although only 31 m apart, were separated by a stream flowing from the Kali River, and thus regarded as two different populations in study period I. In study period II, all sites were at least 94 m apart (Site 7–Site 5: 241 m, Site 5–Site 6: 94 m) and hence treated as three different populations. However, it is unknown whether Site 3–Site 5 and Site 4–Site 6, which are 28 m and 36 m apart in study periods I and II, respectively, belonged to the same population or subpopulations. Hence, data from all seven sampling sites/populations were analysed and presented separately according to their respective study periods to better account for all *R. cantleyi* populations studied.

### Bud monitoring and flowering

*Rafflesia cantleyi* is an obligate endoparasite on the vine of *Tetrastigma rafflesiae* (Vitaceae) (Nais 1997; Wong and Latiff 1994). *Rafflesia* can be found on the roots and climbing stems of its *Tetrastigma* host, with some extensive unobservable roots/stems that may be concealed and linked underground. All above-surface *Rafflesia* flower buds were found by passive search that is, by scrutinizing and removing forest litter in search of other buds whenever a bud was first detected at one site. As such, this may prove to be an inevitable study limitation and underestimate the actual population. However, while there were cases in which some undetected below-ground buds escaped the initial search, when they grew bigger and surfaced above ground later, they were eventually added to the monitoring system.

All live flower buds were monitored almost every fortnight. Each bud was tagged with a reference code containing a plot and bud number. During each visit, the diameter of these live buds was measured using a Vernier calliper (0.01 mm accuracy, Mitutoyo; for bud size less than 10 cm across) or a measuring tape (0.1 cm accuracy; for bud size larger than 10 cm across). The diameter of the buds was taken as the average of the narrowest and broadest measurements made across the buds. The bud development was then categorised into different size classes for further analysis following methods by Nais (2001). This was because no detailed photographs were taken for each bud on the developmental phases unique to *Rafflesia* bud growth (i.e., cupule, bracts and perigone phase), as in Susatya (2020) and Tolod et al. (2021). The collated bud growth and flowering data were initially meant for predicting flowering events to study the diversity of insect visitors and pollinators to *Rafflesia* flowers.

The division of size classes of flower buds for size distribution study varied greatly with *Rafflesia* species and researchers. Size classes with intervals of 2 cm (*R. pricei* and *R. tengku-adlinii*, Nais 2001), 3 cm (*R. keithii,* Nais 2001; *R. bengkulensis*, Susatya et al. 2017) and 5 cm (*R. zollingeriana* Koord.*,* Lestari et al. 2014) were used. The different intervals may be due to the different *Rafflesia* species’ bud and flower sizes. In this study, we chose 5 cm intervals for *R. cantleyi* because, for every increment of 5 cm, the size classes correspond quite closely with *Rafflesia*’s phase of development, although not entirely (as some phases overlapped during the transition phase). For example, the 0–5 cm size class corresponds with the cupule and cupule-bract phase, the 5–10 cm size class corresponds with the bract phase, the 10–15 cm size class corresponds with the bract-perigone phase, and the > 15 cm size class corresponds with the perigone phase for *R. cantleyi*.

The mortality rate was determined by the percentage of dead buds over the total number of buds found at each study site. At the end of the two study periods, the data for flower bud diameter, flowering event and sex of flower were used to determine bud growth rate and fate, size distribution, blooming success and sex ratio of *R. cantleyi* for each population. Additional but not exhaustive observations, such as bite marks, bud dislocation, shrinkage and rotten buds, were also recorded to account for possible causes of bud damage and mortality.

A mature bud is a bud at late perigone phase and usually flowered in the subsequent visit. All buds that successfully bloomed into flowers were included for bud growth rate estimation. The difference between two consecutive measurements of the diameter of all buds at each stage (size classes) was divided by the number of days between the two measurement dates and subsequently averaged to estimate the mean growth rate. The time taken to bloom (days) for all buds was determined by working backwards from the date of floral blooming relative to the date when the first bud diameter measurement was made within a given stage. Specifically, bud growth for three buds found at ca. 2–3 cm across was followed through until floral blooming and used to estimate the duration of the generative stage in the life cycle of *R. cantleyi*.

The sex of the flower was initially determined by inserting a middle finger to touch the underside of the floral disc for the presence of anthers and pollen mush of the blooming flower. It was double confirmed by physical examination of the reproductive organ of the withered flower in the subsequent visit. The withered *Rafflesia* usually remained intact for months, despite the whole flower turning black. The duration of the life cycle of *R. cantleyi* was estimated based on the growth data obtained for the generative stage in this study and extrapolated from literature on the fruit development and vegetative stage of other *Rafflesia* species (Beaman and Adam 1984; Meijer 1997; Nais 2001; Tolod et al. 2021; Whitten et al. 1996).

### Statistical analysis

Before analysis, all data were subjected to the Shapiro–Wilk Normality test (*P* =  0.05) and the equal variance Test (*P* = 0.05). To understand whether the size of mature buds was influenced by different sites, the mature bud diameter between sites in each study period was compared using one-way analysis of variance and the means separated by Tukey’s test (*P* = 0.05). A Chi-square test (*P* = 0.05) was used to determine whether the sex ratio of the *R. cantleyi* flowers of a given population deviated significantly from the 1:1 ratio. Means of female and male bud growth rate and time to blooming for different size classes were analysed and compared by Student’s *t*-test or Mann–Whitney *U*-test when data were not distributed normally. Non-linear regression was used to obtain exponential growth model equations and coefficient of determination (*R*^2^) for selected young male and female buds (< 6 cm in diameter) from different plots that successfully bloomed. All statistical analyses were performed using Sigma Plot 12.0.

We employed a generalized linear mixed model (GLMM) (lme4 package, version 1.1–34) to assess the effect of different sites (site as a random effect) on the growth of flower buds that developed into mature buds before blooming in each study period. Monthly temperature and rainfall data during the study periods were obtained from the nearby (within 36 km radius) meteorological station, Batu Embun (3° 58' N 102° 21' E). Kendall’s Tau correlation analysis was performed to determine the relationship between flowering phenology of *R. cantleyi* to rainfall and temperature at *P* = 0.05. Both the GLMM and Kendall’s Tau correlation analyses were executed within the R environment (version 4.3.1).

## Results

A total of 247 buds at various stages were found and monitored during the two study periods [study period I (Dec 2009 to February 2013): 140 buds; study period II (May 2016 to June 2018): 107 buds] in Lata Jarum, Raub, Pahang (Table 2). All the buds/flowers found were on the ground (horizontal vines) except for one climbing vine with seven buds in study period II.

**Table 2:** The fate of all *Rafflesia cantleyi* buds monitored in seven sites in two separate study periods at Lata Jarum forest reserve, Raub, Pahang, Peninsular Malaysia

Site	Monitoring period	Total buds	Missing (%)	Herbivory (%)	Mortality (%)	Bloomed (%)	Still in bud (%)
Study Period I							
1	30 Dec 2009–19 May 2011	29	6.9	0.0	69.0	24.1	0.0
2	20 Nov 2009–13 Feb 2013	41	0.0	0.0	56.1	39.0	4.9
3	20 Nov 2009–13 Feb 2013	31	3.2	0.0	71.0	25.8	0.0
4	12 Dec 2009–13 Feb 2013	39	2.6	2.6	53.8	41.0	0.0
Subtotal		140	2.9	0.7	61.4	33.6	1.4
Study Period II							
5	03 May 2016–23 April 2018	13	0	0.0	76.9	23.1	0
6	03 May 2016–04 June 2018	78	6.4	2.6	29.5	48.7	12.8
7	19 Aug 2016–04 June 2018	16	0	6.3	37.5	56.3	0.0
Subtotal		107	1.7	2.8	36.4	46.7	9.3

### Size distribution, fate of buds and sex ratio

- ***(i) Study period I***

**Fig. 3:**
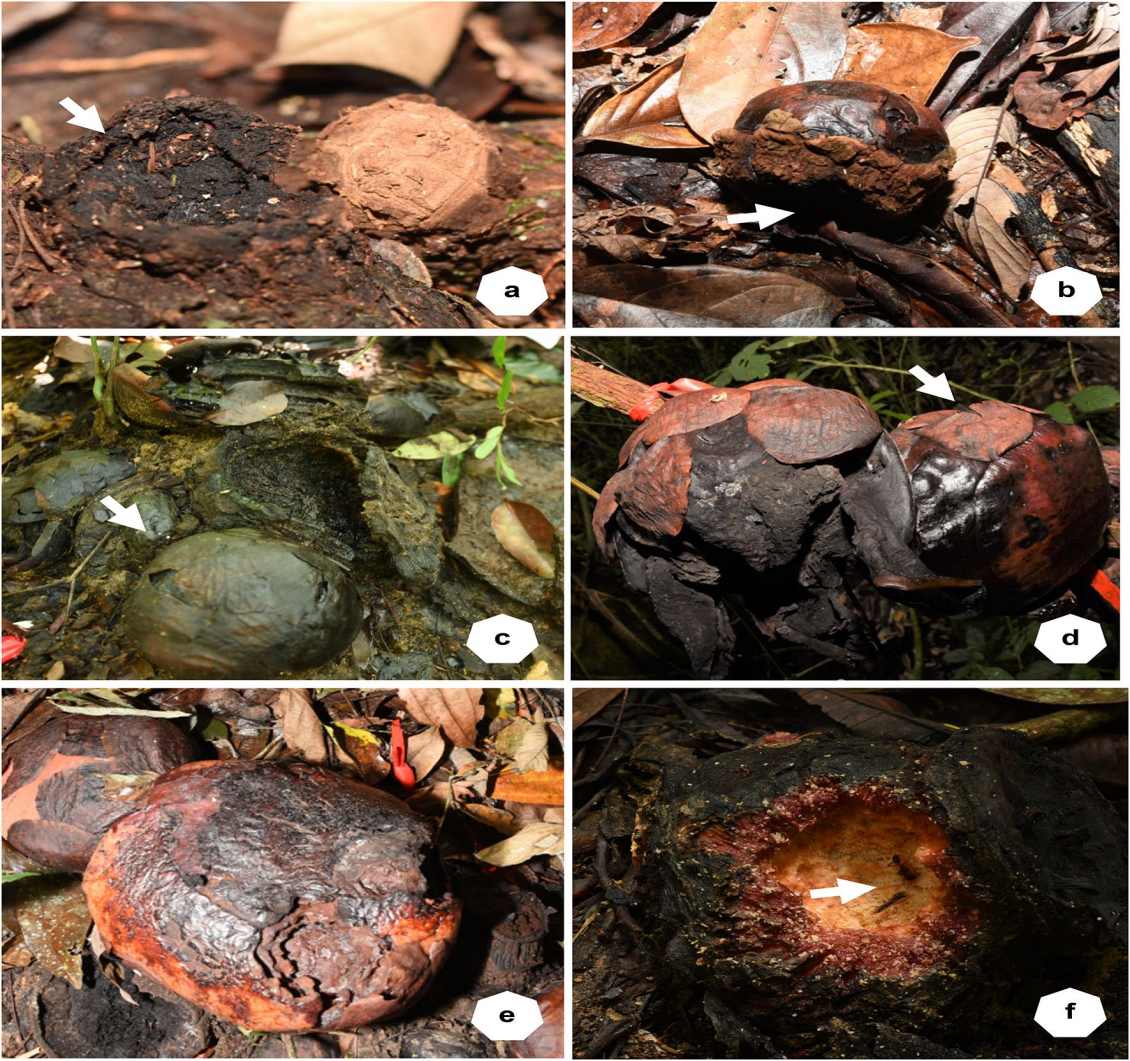
Various causes of mortality in *Rafflesia cantleyi* development stage: (**a**) a long-dried bud at cupule stage (arrow) beside a healthy bud at the post-emergence stage; (**b**) a dislodged bud at the bract stage separated from the host vine (arrow) caused by roaming wild boars; (**c**) rotten buds at bract; (**d**) one aerial mature bud died at perigone stage; (**e**) a bud at the perigone stage was bitten by an animal and failed to bloom; (**f**) a mature fruit showed signs of herbivory, possibly by a wild boar, and attended by ants (arrow)

**Fig. 4:**
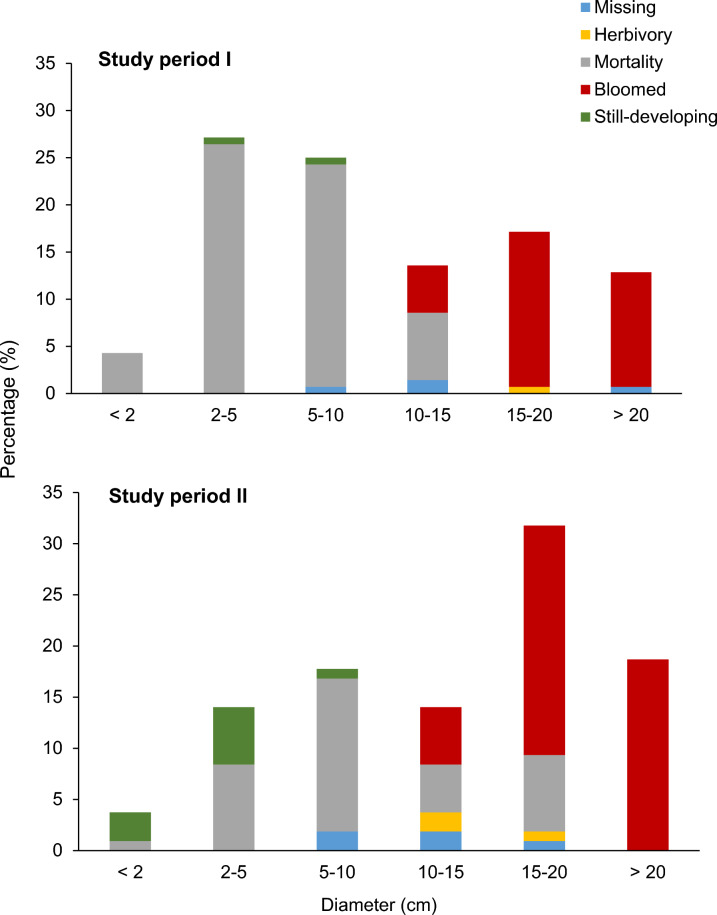
The fate of *Rafflesia cantleyi* according to bud size class distribution (in diameter, cm) at Lata Jarum forest reserve, Raub, Pahang, Peninsular Malaysia from two different study periods, study period I (December 2009–February 2013) and study period II (May 2016–June 2018)

Four populations, each consisting of 29 to 41 buds, were monitored (Table 2). The mortality rate of buds was high, with an average of 61.4%. The symptoms of bud mortality were common across the four sites: either shrinkage or rot (Fig. 3a, c). Mortality of flower buds varied with sites, ranging from 53.8 to 71.0%, with Sites 1 and 3 recording the highest mortality rate: 69–71% (Table 2). Further analysis showed that the highest mortality rate occurred in buds smaller than 5 cm across (30.7%), followed by those 5–10 cm across (23.6%) (Fig. 4). The mortality rate decreased once the buds reached 10 cm in diameter (7.1%; Fig. 4). A low percentage (2.9%) of buds were found missing throughout study period I (Table 2) with apparent cut wounds on the host vine. Most of the missing buds were around 10 cm in diameter, except for one at 20.1 cm. In addition, one bud (0.7%) with the size of a mature bud (diameter 18.1 cm) that failed to bloom was found with bite marks, possibly by wild animals (Fig. 3e). Toward the end of study period I, the once productive site had a low number (1.4%) of still-developing buds.

**Fig. 5:**
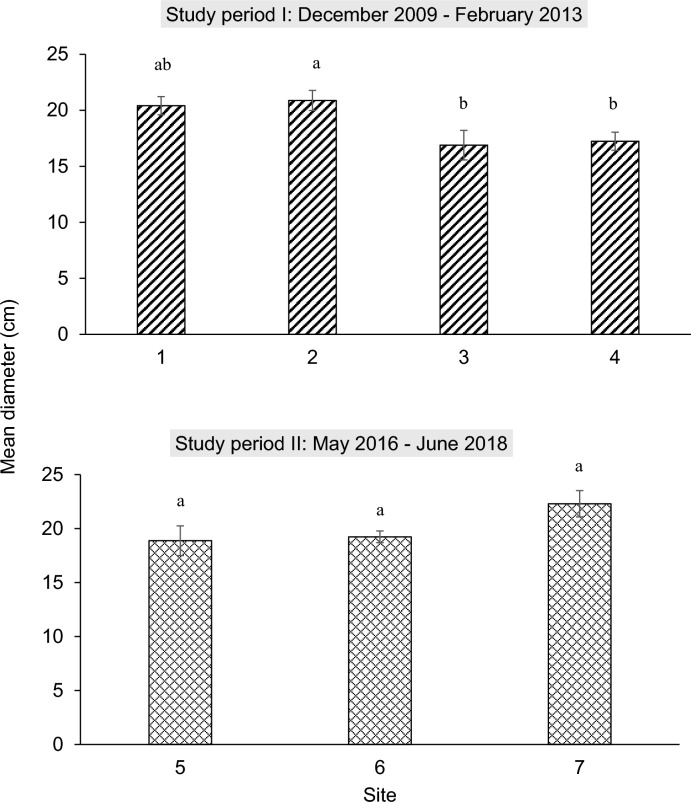
Mean diameter (± SEM; cm) of *Rafflesia cantleyi* mature buds from different sites during the two discrete study periods at Lata Jarum, Raub, Pahang: (**a**) study period I, December 2009- February 2013 and (**b**) study period II, May 2016–June 2018. Letter designations shown indicate significant statistical differences at *P* = 0.05 (Tukey’s Test)

The size of the mature buds varied significantly between the four sites in study period I (*F* = 4.577, *df* = 3, 40; *P* = 0.008). The average diameter of mature buds at Site 2 (20.9 ± 0.9 cm) was significantly larger than those at Sites 3 and 4 (*P* < 0.05, Tukey’s test); no significant difference was found between Site 1 (20.4 ± 0.8 cm) and all other sites (*P* > 0.05, Tukey’s test; Fig. 5). The largest mature bud measured was 27.1 cm in diameter (Site 2), while the smallest was 12.9 cm (Site 4). The average diameter of the mature bud was 18.93 ± 0.55 cm.

Of the 140 buds, 33.6% successfully reached maturity and bloomed. Populations at Sites 2 and 4 recorded the highest blooming successes (39.0% and 41.0%, respectively), followed by Site 3 (25.8%), while Site 1 had the lowest floral blooming success (24.1%; Table 2). Flowers that bloomed during study period I were primarily male flowers except for Site 3, and when the results were combined, they gave a significant male-biased sex ratio of 1: 4.88 (χ^2^ = 20.45, *P* < 0.001; Table 3).

- ***(ii) Study period II***

**Table 3:** The sex ratio of *Rafflesia cantleyi* flowers of seven site bloomed in two separate study periods in Lata Jarum, Raub, Pahang

Study period	Site	Total bloomed	Female (F)	Male (M)	Sex ratio (F:M)	χ^2^	*P*
I	1	7	0	7	0:7	7.00	< 0.01
	2	16	0	16	0:16	16.00	< 0.001
	3	8	7	1	7:1	4.50	< 0.05
	4	16	1	15	1:15	12.25	< 0.001
	Subtotal I	47	8	39	1: 4.88	20.45	< 0.001
II	5	3	1	2	1:2	0.33	> 0.05
	6	38	37	1	37:1	34.11	< 0.001
	7	9	6	3	6:3	1.00	> 0.05
	Subtotal II	50	44	6	7.33: 1	28.88	< 0.001

Study period I: Dec 2009–Feb 2013; study period II: May 2016–June 2018

The average bud mortality in study period II was relatively low (36.4%) compared to bud mortality in study period I. Of these, the population at Site 5 recorded the highest mortality at 76.9%, while Sites 6 and 7 recorded 29.5 and 37.5%, respectively (Table 2). Further analysis showed that bud mortality according to size classes, in decreasing order, was 5.0 < X ≤ 10.0 cm (15.0%), followed by < 5 cm (9.3%), 15–20 cm (7.5%) and 10.0 < X ≤ 15.0 cm (4.7%) (Fig. 4). While 4.7% of buds monitored were missing during study period II, some buds with diameters between 10 and 20 cm showed evidence of herbivory (2.8%) (Fig. 4). Upon careful examination, the causes of bud destruction were animal herbivory (Fig. 3e, f) and dislodgement from the host vine by roaming wild boars (Fig. 3b). One aerial mature bud, by the site of a bloomed flower, was found dead (Fig. 3d). Towards the end of study period II, about 9.3% of buds at various stages of development remained (Table 2).

There was no significant difference in mature bud size between the three populations monitored (*F* = 0.212, *df* = 2, 38; *P* = 0.123; Fig. 5). The smallest and largest mature buds of *R. cantleyi* were 13.2 cm and 25.25 cm, respectively. The average diameter of a mature *R. cantleyi* bud in study period II was 19.59 ± 0.50 cm.

Of the 107 buds monitored across three sites in study period II, 46.7% successfully bloomed. The population at Site 5 had the lowest blooming success (23.1%), followed by Site 6 (48.7%), while Site 7 had the highest successful bloom percentage (56.3%) (Table 3). As for the only climbing (aerial) vine found in Site 5, out of the seven buds grown on this vine, four buds successfully developed into mature buds and bloomed. Sites 6 and 7 produced significantly more female than male flowers, with the overall bloomed flowers biased towards the female sex (7.33:1; χ^2^ = 28.88; *P* < 0.001; Table 3).

### Bud growth

**Fig. 6:**
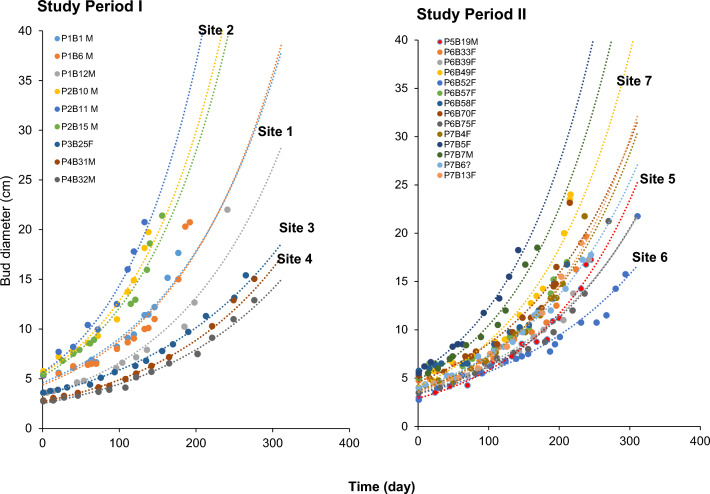
Graphs depicting the exponential growth pattern of *Rafflesia cantleyi* buds from different sites monitored as small buds (< 6 cm) to flowering during the two study periods: I (Dec 2009–Feb 2013; 39 months) and II (May 2016–June 2018; 26 months). Nine and 14 buds were monitored during study periods I and II, respectively. Letters P and B denote plot and bud while M and F, male and female flowers, respectively

Based on the mean growth rate of various bud size classes, *R. cantleyi* flower bud growth followed an exponential pattern (Table 4; Fig. 6). However, it was apparent that each bud had a specific growth curve (Fig. 6). It was also evident that the growth profiles for buds within the same site clustered together regardless of the sex of the flowers, particularly when the sites are distantly located or with different physical attributes (Fig. 6; also refer to Table 1). The GLMM analysis showed that sites exerted a significant influence on the growth of *R. cantleyi* bud diameters in study periods I (*P* < 2.2 × 10^–16^) and II (*P* < 4.5 × 10^–6^).

**Table 4:** Estimated mean female and male *Rafflesia cantleyi* bud growth rates and time to bloom according to diameter class

Diameter class, X (cm)	Mean growth rate (± SD; cm day^−1^)				Mean time to bloom (± SD; days) (min–max)			
	*n*	Female	*n*	Male	*n*	Female	*n*	Male
2.7 < X ≤ 5.0	18	0.0102 ± 0.0014a	12	0.0109 ± 0.0018a	18	249.0 ± 34.8a (191–313)	12	235.5 ± 39.0a (181–291)
5.0 < X ≤ 10.0	67	0.0384 ± 0.0124a	63	0.0480 ± 0.0148b	67	143.3 ± 43.1a (71–230)	63	113.6 ± 32.5b (55–185)
10.0 < X ≤ 15.0	52	0.1218 ± 0.0683a	37	0.1162 ± 0.0430a	52	49.4 ± 18.8a (11–92)	37	48.7 ± 17.3a (23–93)
15.0 < X ≤ 20.0	23	0.3297 ± 0.2108a	25	0.2996 ± 0.1551a	23	20.7 ± 12.4a (5–62)	25	21.7 ± 11.4a (9–47)
X > 20.0	11	0.8955 ± 0.5704a	11	0.7773 ± 0.4450a	11	12.6 ± 15.3a (3–45)	11	8.5 ± 4.4a (3–15)

Means between sexes with different alphabets are significantly different at *P* = 0.001 (Student’s *t*-test or Mann–Whitney *U*-test when the normality of data is not met)

Table 4 shows the mean growth rate and time to bloom for buds of different size classes. The growth rate of both male and female buds at the early development stage (≤ 5 cm in diameter) was relatively consistent and slow, about 0.1 mm daily, with no significant difference between sexes (*P* > 0.05). The growth rate was also slow for bud size class 5.0 cm < X ≤ 10.0 cm, approximately 0.38–0.48 mm day^−1^. However, the mean growth rate of female buds was significantly slower than that of male buds at this stage, resulting in a significantly longer time to bloom (*P* < 0.001, Mann–Whitney *U*-test; Table 4). The growth rate of bud size class 10.0 cm < X ≤ 15.0 cm gradually increased to ca. 1.1 to 1.2 mm day^−1^ without any significant difference between male and female buds. An accelerated growth rate (about 3–8 mm day^−1^) was apparent when buds grew beyond 15 cm in diameter; however, the growth rate and time to bloom were also highly variable at this stage (Table 4).

### Flowering and phenology

**Fig. 7:**
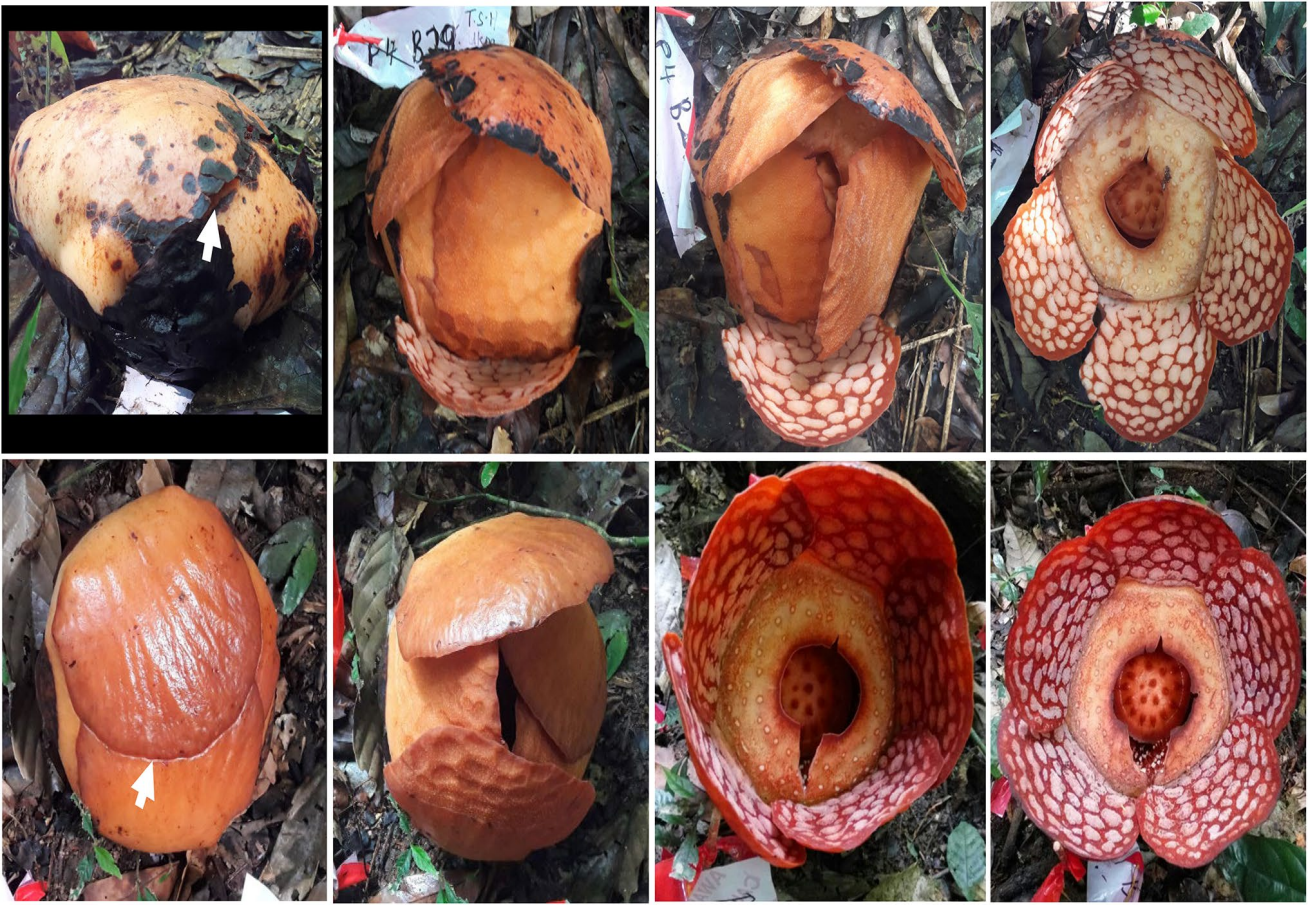
Two types of blooming mechanisms were observed for *Rafflesia cantleyi*. Top row: ‘flap-type’—when the outermost perigone lobe covers almost the entire top surface of whole flower bud. During flower blooming, the outermost perigone lobe first lifted up (arrow), followed by the one below, and so on until the last perigone lobe is opened. Bottom row: ‘spiral-type’—outermost perigone lobe partially covers about midline of the mature flower bud (arrow). All the perigone lobes open almost simultaneously during flowering

We observed two types of flowering pattern in *R. cantleyi* related to the morphology of mature flower buds at their late perigone phase (near to flower blooming). The ‘flap-type’ referring to the outermost perigone lobe being a single flap covers almost the entire top surface of whole flower bud (Fig. 7). During flower blooming, the perigone lobes will open one by one: the outermost perigone lobe will be the first to lift up, followed by the one below, and so on until the last perigone lobe is opened (Fig. 7). The second ‘spiral-type’ which was said to be rarer, referring to the partial coverage of the outermost perigone lobe up to the midline of the mature flower bud (Fig. 7). When it is time to bloom, all the perigone lobes open almost simultaneously (Fig. 7).

**Fig. 8:**
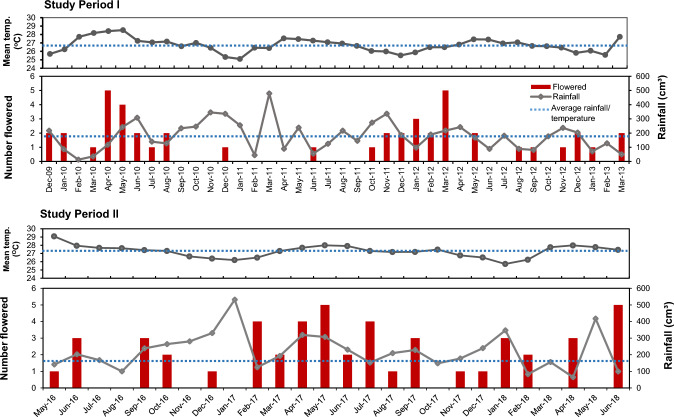
The flowering phenology of *Rafflesia cantleyi*: monthly total number of flowers bloomed in relation to monthly rainfall in Lata Jarum, Raub, Pahang throughout the two study periods: I (39 months; December 2009 to February 2013); II (26 months; May 2016 to June 2018)

*Rafflesia cantleyi* was observed to flower year-round at Lata Jarum, with slightly more blooms occurring from April to June, except during 2011–2012. However, it did not appear seasonal, as evident by the lack of synchronisation and consistent flowering patterns between the two studies (Fig. 8). The blooming of *R. cantleyi* buds showed no significant correlation with rainfall (study period I: ${{\text{r}}}_{\uptau }=-0.02$; study period II: ${{\text{r}}}_{\uptau }=-0.07$) and temperature (study period I: ${{\text{r}}}_{\uptau }=-0.01$; study period II: ${{\text{r}}}_{\uptau }=0.08$) (Kendall’s Tau correlation, *P* > 0.05).

**Fig. 9:**
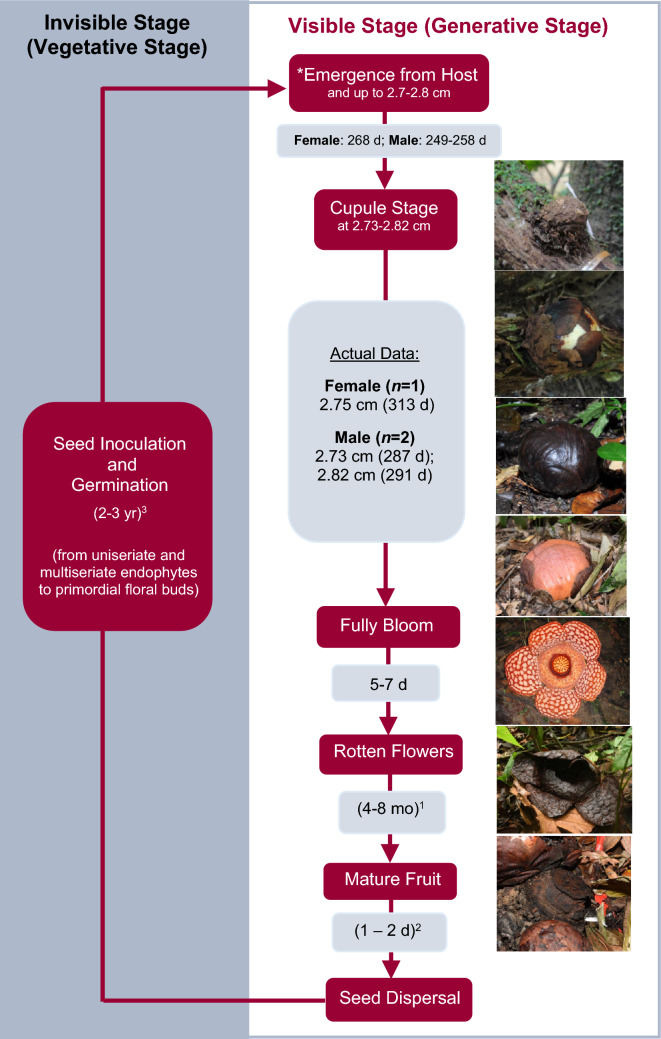
Reconstructed life history of *Rafflesia cantleyi* based on actual developmental data, estimated data from extrapolated bud growth below diameter 2.7 cm (based on bud growth rate for bud size 2.7 − 5 cm in this study) and literature. Length of times in parentheses are reported cases from literature for other species: ^1^Beaman and Adam 1984, Whitten et al. 1996, Meijer 1997, Tolod et al. 2021; ^2^Nais 2001; ^3^Meijer 1997, Hidayati et al. 2000

Based on the data, fewer flowers bloomed when the preceding months received lower-than-average monthly rainfall. Conversely, more flowers bloomed when the preceding months received higher-than-average monthly rainfall during the Northeast monsoon, although the pattern is inconsistent throughout the study periods (Fig. 8). From our field observation, the buds grew faster during the rainy season, which may lead to more blooming in later months. Surrounding air temperature also did not appear to affect *R. cantleyi* flower phenology. Depending on weather conditions, a full bloom *R. cantleyi* flower may last for five to seven days before the whole flower completely turns black (Fig. 9). Flower withering is accelerated under wetter or rainy weather but prolonged under drier conditions.

## Discussion

The present study is the first long-term monitoring report on the size distribution, bud growth, flowering phenology and life history of *R. cantleyi*. The 65-month monitoring studies from two discrete periods successfully revealed much of the dynamic nature of the size distribution and flowering phenology of *R. cantleyi*. This valuable information has shed more light on the secret life of the gigantic and parasitic flowers.

### Size distribution, fate of buds and sex ratio

The bud size distribution of *Rafflesia* is dynamic in nature and changes from time to time. Our observations in both studies showed that a productive *Rafflesia* population often consisted of buds at various growth stages, and the distribution of bud size classes was dynamic over time. The composition of new, developing and mature buds, and blooming of *R. cantleyi* within a subpopulation/population at a particular time frame may indicate its overall development stage, whether it is a young, maturing or old population. For example, a young *R. cantleyi* population would have floral buds skewed toward those < 10 cm in diameter and vice versa. Towards the end of the study, the sites were mainly left with a few developing buds, remains of withered flowers or few fruit sets.

Categorising bud mortality according to size classes and mortality rates and identifying possible causes may help pinpoint the vulnerable stage and main factors contributing to high mortality within a *Rafflesia* population. The causes of floral bud mortality are generally high/low soil moisture, trampling or dislodgement by humans/animals, herbivory and natural disasters to varying degrees depending on locality (Emmons et al. 1991; Kusuma et al. 2018; Lestari et al. 2014; Meijer 1985).

In Lata Jarum, young *R. cantleyi* buds < 5 cm in diameter are the most vulnerable development stage, followed by those 5–10 cm in diameter. It was observed that the high mortality rate of young buds was due to natural death and was site-specific. The bud growth profiles clustered according to sites further attested to this observation. We speculate that the water and nutrient availability to host vines may potentially affect the growth and survival of flower buds given the holoparasitic nature of *Rafflesia*, which obtain all nutrient requirements, including water, from its host vines (Barkman et al. 2004; Davis and Wurdack 2004; Nais 2001). Soil texture, soil structure and plant rootability are crucial factors in determining the amount of soil water available for plants (de Jong van Lier 2014). Hence, the influence of soil physics and moisture content in relation to rainfall on the growth and survival of *Rafflesia* buds certainly warrant future investigation.

A high mortality rate of buds is typical for almost all *Rafflesia* species and populations*.* Bud mortality at a rate of 50–60% has been reported for *R. arnoldii,* *R. keithii, R. patma* and *R. tengku-adlinii* (Hidayati et al. 2000; Meijer 1958; Nais 2001). A mortality rate as high as 93–100% has also been reported for *R. pricei* and *R. bengkuluensis* (Beaman et al. 1988; Susatya et al. 2017; Susatya 2020). Nais (2001) suggested that high bud mortality may also be a form of natural population control due to the inability of host plants to cope with the increase of bud total mass and subsequent nutrient demand. A study on the number of bud load per host on the population attributes of *R. bengkuluensis*, Susatya et al. (2017) lend some support to the host internal control hypothesis. They showed that each subpopulation of *R. bengkuluensis* consisted of an average of 4.75 buds and never exceeded 8 buds, with 5 out of the 11 subpopulations recording 100% mortality and an average of 65% mortality for the entire population. In this study, there was no attempt to map bud load per host, as the host vines were mostly concealed beneath the soil, making tracing difficult and potentially causing bud injury. Therefore, we could not provide additional data to test or verify the relationship of bud load per host plant and bud mortality due to nutrient demand. The hypothesis remains to be tested and verified for *R. cantleyi* and other *Rafflesia* species.

High mortality rate of young buds was also reported to be associated with the host immune response (Nikolov et al. 2014a). Recent histological work on the host-endophyte interface further revealed that the vegetative body of *Rafflesia* individuals resides within the hosts in a form of microscopic uniseriate filaments (Bascos et al. 2021; Mursidawati and Wicaksono 2020; Nikolov et al. 2014a). The uniseriate filaments apparently grow rather slowly and would remain undetected as pathogen invasion by hosts (Nikolov et al. 2014a), presumably undifferentiated from host cells or less significant to the host growth and nutritional distribution until they enter the generative stage and form protocorm, the primordial flower bud. When the parasite grows significantly during bud expansion, the host respond by clogging its own vessels with mucilage at the base of the protocorm which eventually caused bud mortality (Nikolov et al. 2014a).

Our study showed that mortality of buds greatly reduced when they grow larger than 10 cm in diameter, corresponding with the early perigone stage. When developing buds enter differentiation stage which lead to floral organ formation, they start compressing the host vascular tissue surrounding the flower buds. Mursidawati and Wicaksono (2020) have demonstrated how *Rafflesia* strategise by adapting its growth mechanism which minimize the host vasculature damage, i.e. inhibits only the growth of one or two host vascular bundles, when one single flower bud or multiple flower buds are growing the same direction in a series. When multiple buds grow in different angles/directions within a vine, it would affect more host vascular bundles surrounding the flower buds and will causes more damage to the host, leading to possible death in the future or eradication of a single population within a single host, which will be costly to the survival of the parasites (Mursidawati and Wicaksono 2020).

Young buds dislodged by roaming wild animals, animal herbivory, and possible bud collection by nearby indigenous people amounted to less than 5% in Lata Jarum, Raub. This percentage is considered low compared to other *Rafflesia* species reported in Sabah Borneo (6.7%; Nais 2001) and Java (10.65%; Kusuma et al. 2018). There are a few ethnic groups of indigenous people (known as ‘orang asli’ in local language) living in villages near the forests, such as Kampung Orang Asli Yol, Ulu Dong district, Lata Jarum (Er et al. 2012). The indigenous people in Malaysia and Indonesia often harvested *Rafflesia* buds for their purported medicinal properties (Nais 2001; see also review by Wicaksono et al. 2016). *Rafflesia* floral bud extracts were believed to able to stop internal bleeding, accelerate the healing of the uterus and restore body figure after childbirth (Nais 2001) as well as an aphrodisiac for men (Wong and Latiff 1994). The latter was supported by a recent study by Sumaia et al. (2015) whereby the extract of *R. cantleyi* buds was shown to act as an aphrodisiac by increasing testosterone level and enhanced sexual behaviour in male rats.

In the present study, bud herbivory was mostly observed for flower buds measuring 5–15 cm in diameter (bract to perigone stage). However, buds larger than 15 cm across (perigone stage) could also be the target of animal herbivory. In one case, the damage was severe, causing the close-to-bloom bud to fail to bloom. Apart from bud herbivory, fruits may also be subjected to animal frugivory. Such frugivore-damaged fruits were also visited by ants as observed in this study. Therefore, depending on the stage of fruit and seed maturity, animal frugivory may aid in *Rafflesia*’ seed dispersal and inoculation on *Tetrastigma* hosts.

The successful blooming rates for the *R. cantleyi* populations studied varied greatly with sites which seemed to correspond well with the results of bud mortality versus sites; sites with a low blooming rate had a high mortality and vice versa. The blooming rates of *R. cantleyi* at Lata Jarum were relatively high compared to other *Rafflesia* species, for example: *R. adlinii* (2.8%), *R. pricei* (9.1%; Nais 2001), *R. zollingeriana* (7.89%; Lestari et al. 2014) and recently, *R. consueloae* (19.73%;  Tolod et al. 2021). The increase in blooming success in study period II may be attributed in part to the yearly closure (November to January) of all recreational forests beginning in 2014 by the Department of Forestry, Pahang. This short-term closure of recreational forests to the general public serves two purposes: first, forest health maintenance and second, as a precautionary measure against natural disasters during monsoon periods, which coincides with the highest monthly rainfall recorded during the Northeast monsoon (October to March). This measure may have reduced human disturbance and left all flora and fauna undisturbed in their natural habitats, including the *Rafflesia* population, thus contributing to the increase in bud survival and flowering rates observed in study period II.

Male-biased *Rafflesia* populations have been widely reported in the literature (Bänziger 1991; Galang 2007; Meijer and Elliot 1990; Nais 2001; Yahya et al. 2010). Female flowers are rare in most of the *Rafflesia* population studied. There has been no report on the simultaneous blooming of female and male flowers within a population, including this study, which may further contribute to *Rafflesia*’s rarity (Nais 2001). There has also been no report on a single *Rafflesia* population dominated by female flowers. This phenomenon may be partly due to most of the previous ecological studies taking place over a short period of time, ranging between one and eight months (Hidayati et al. 2000; Lestari et al. 2014; Susatya et al. 2017; Susatya 2020), except for *R. keithii* and *R. pricei*, which were studied for 20–29 months by Nais (2001). Moreover, a re-visitation of the same *Rafflesia* study site has rarely been conducted or reported in the literature.

For the first time, the present study reported that female flowers could also dominate a *Rafflesia* population. The long-term nature of the present study, which spanned almost ten years (2009–2018), though with a gap of 2–3 years between the two discrete monitoring periods, was instrumental in this finding. Female flowers dominated in two of the seven sites monitored, while three out of seven sites were dominated with male flowers. This study, however, reaffirmed that a skewed sex ratio is common in *Rafflesia* populations. The reason behind single-sex flowers dominating flowering events in the sexual reproduction of this plant species is not well understood. In general, mechanisms of sex determination in plants could be related to chromosomal, genetics, phytohormonal and environmental factors (water availability, soil physicochemical conditions, etc.) associating with the transition from vegetative to reproduction (Irish & Nelson 1989; Loehwing 1938; Milewicz and Sawicki 2012; Pannell 2017). However, the factors affecting sex determination in *Rafflesia* and its mechanisms are largely unexplored. Since all buds and flowers were not traced to plant clones or host individuals in this study, we cannot rule out entirely the possibility of some large female *Rafflesia* clones dominating a population. Barkman et al. (2017) reported that numerous plant clones can infect a single vine but an individual *R. cantleyi* clone could also produce up to 25 flowers in the same host. Considering the endoparasite’s cryptic life history, future study employing least-damaging sampling of tissue from flower buds and DNA-based clone identification for assigning each *Rafflesia* flowers to an individual clone could shed more light on this phenomenon.

### Bud growth

An exponential growth pattern of the generative stage was first documented for *R. patma* based on composite data obtained from a six-month study (Hidayati et al. 2000). Similar observations were also recorded for other species in Rafflesiaceae: *R. arnoldii* (Meijer 1958), *R. kerrii*, *Rhizanthes zippelii* Blume (Spach) and *Sapria himalayana* Griffith (Bänziger 1991, 1995; Bänziger et al. 2000). In a recent survey, Susatya (2020) meticulously divided all bud growth stages according to different morphological states and bud diameters to document the life history of *R. arnoldii*. An exponential bud growth equation was yielded and proposed to be a species-specific model that is useful in predicting age and flowering events of *R. arnoldii* based on physical bud measurements.

*Rafflesia cantleyi* showed a similar exponential growth pattern, that is, its growth rate was slow for young buds but increased drastically and exponentially at the perigone stage before flowering. The mean growth rate of flower buds below 5.0 cm was the slowest, collaborated with the growth rate at cupule stage reported for *R. arnoldii* and *R. patma* (Hidayati et al. 2000; Susatya 2020), followed by size classes 5–10 cm (corresponded to bract stage), 10–15 (corresponded to bract-perigone state), 15–20 (corresponded to perigone stage) and > 20 cm (perigone stage). Additionally, by comparing the average bud growth rate at different size classes, we further showed that the female buds had a significantly slower growth rate than the male buds when the flower buds were between 5 and 10 cm across. The direct field observation for the growth duration of a female bud with diameter 2.75 cm (313 d) to flowering was approximately a month slower than the two male buds observed, i.e. 2.73 (288 d) and 2.82 cm (291 d) which further supported this result. This finding also collaborated with those of *R. arnoldii,* that the development time of a female flower is estimated to be slightly longer than that of a male flower (Susatya 2020).

We also showed that the exponential equations obtained are unique to sites. We found that buds from the same plots demonstrated similar growth rates and their profiles tended to cluster together, regardless of sex, and corresponded with the results obtained for differences in mature bud size between sites. These findings support our argument that site-specific factors, such as soil physics and moisture content, may potentially affect the growth rate, mortality and flowering success of *R. cantleyi*. It also suggests that the growth model of flower buds may not be species-specific as proposed by Susatya (2020) but rather highly associated with sites. Susatya (2007) also proposed that different sites and hosts could impact the bud growth rates of *R. arnoldii*. Unfortunately, soil samples and their moisture content were not sampled and analysed in this study. Hence, future studies on the impact of physical attributes, such as soil type, soil moisture content, distance from water bodies, and slope inclination, are warranted on the overall health, growth rate, and fate of *Rafflesia* populations. This information would shed light and provide further information in the conservation of *Rafflesia*, be it *in-situ* or *ex-situ*.

The mechanisms and pathways of controlling growth and differentiation of *Rafflesia* is largely unexplored until recently. Lee et al. (2016) identified the genes potentially involved in the regulation of growth and development of *R. cantleyi* via comparative transcriptomic analyses on the floral bud and flowers. Further advanced transcriptome analysis revealed that the differentially expressed genes (DEGs) are involved in various phytohormone signal transduction events, such as auxin and auxin transport, cytokinin and gibberellin biosynthesis (Amini et al. 2019). These results imply that transcription factors and phytohormone signalling pathways play a significant role in *Rafflesia* floral bud development. In a preliminary study via an in vivo injection of auxin and cytokinin into *R. patma* flower buds, Mursidawati and Wicksono (2021) have demonstrated that, auxin and not cytokinin is more likely responsible in the growth and differentiation of *Rafflesia* flower buds.

### Flowering and phenology

The flowers of *R. cantleyi* displayed two different flowering mechanisms, i.e. the ‘flap-type’ and the ‘spiral-type’. We were unable to provide an account of flap-type over the spiral-type flowering events in the whole study because we did not have an exhaustive photographic record of the late perigone phase of all mature buds. Although the monitoring was scheduled bi-weekly, some mature buds bloomed unexpectedly a day or two before the scheduled visit (as alerted by the forest rangers). In addition, not all photographs of the buds were taken from top-view that enable a more accurate determination of the position of outer perigone lobe and the second perigone lobe prior to bloom. Nevertheless, we were unsure the factors behind the different flowering mechanism and whether it has any ecological or biological significance in the reproductive ecology of *Rafflesia*.

The seasonality of the *Rafflesia* flowering phenology was not well understood. One of the limiting factors contributing to this gap in knowledge is the research commitment and funding needed for such long-term ecological study in the often-remote habitats of *Rafflesia* populations. Previous ecological studies cited mixed views on the seasonal and non-seasonal nature of flowering phenology in *Rafflesia* spp. In the present study, the flowering phenology of *R. cantleyi* showed a lack of synchronisation and consistency with climatic condition, i.e. temperature and rainfall data, pointing to non-seasonality. This observation of *R. cantleyi* is similar to other *Rafflesia* species in Sabah Borneo (*R. keithii* and *R. pricei*; Nais 2001) and Indonesia (*R. arnoldii* and *R. patma*; Justesen 1922; Hidayati et al. 2000; Meijer 1958) which also displayed non-seasonal flowering phenology. The results confirmed our speculation that only there is a distinct hottest/coldest and dry season that a *Rafflesia* species may demonstrate seasonal flower phenology, such as in the cases of *R. kerii* in southern Thailand and Philippine *Rafflesia* species (Bänziger 1991; Barcelona et al. 2009; Meijer and Elliot 1990; Tolod et al. 2021).

Precipitation in the form of rain may influence the growth rate of the flower buds of *R. cantleyi.* Based on our field observations, flower buds grow quicker during the rainy season, which may lead to more flower blooming in later months. Water absorption by soil and vine hosts may also be affected by other factors such as soil characteristics, nutrients, vegetation and root systems (de Jong van Lier 2014). The relationship of *Rafflesia* flowering phenology to soil physicochemical properties, and rainfall on the growth and survival of *Rafflesia* buds certainly warrants future investigation.

Apart from climatic condition, the flowering phenology of *R. cantleyi* may also be affected by nutritional condition of its host, *T. rafflesiae*. Although data on the flowering, fruiting and nutritional status of *T. rafflesiae* may not be available from this study, it is predicted that immediately after *Tetrastigma* host has flowered and fruited, internal nitrogen level would have been drastically reduced, as nitrogen availability directly contributed to vegetative plant growth and reproduction (Carranca et al. 2018). This may also indirectly limit *Rafflesia* plant’s nitrogen uptake and subsequently affect growth and flowering pattern. The knowledge of the flower phenology of *Rafflesia* blooming is vital for conservation purposes. For example, restricting site visitation to the peak flowering season could potentially reduce the chances of bud trampling by otherwise unplanned and frequent visits, especially when no proper conservation measures are in place.

### Reconstructed life history

One of the many factors contributing to the rarity of *Rafflesia* is that the endoparasite has a cryptic and long development period. Thus, documenting its complete life history, particularly the vegetative stage, have proven challenging to study directly.

While seed inoculation and germination remain a mystery, recent histological studies have revealed much about the parasite-host interactions after germination. The work on different *Rafflesia* species have consistently shown that infection begins within the vascular cambium (Bascos et al. 2021; Mursidawati et al. 2019; Wicaksono et al. 2021) where the uniseriate endophyte may reside in its vegetative form prior to radial spread to the vascular tissue for more than half a year before reproductive stage (Bascos et al. 2021). The infected host may not show any sign of being infected outwardly, hence the duration estimation of this stage may be a great challenge. Subsequent growth of the endophyte leads to lateral spread toward the xylem and phloem, developing to multiseriate clumps before giving rise to the incipient floral shoot, the onset of the generative stage (Bascos et al. 2021; Mursdawati et al. 2019; Nikolov et al. 2014a, 2014b; Wicaksono et al. 2021).

At the early stage of emergent, the endophytic parasite appears as an irregularity or unnoticeable swelling in the host bark (Tolod et al. 2021). To estimate the total development time required for the buds to develop from an unnoticeable swelling (emergence stage) to the smallest bud size detected in this study (female bud: 2.75 cm; male bud: 2.73 cm), we had assumed the growth rate for buds smaller than 2.7 cm across is similar to bud diameter between 2.7 < X ≤ 5.0 cm. This approach may have overestimate the growth rate of an unnoticeable bud, as the growth rate at this stage is slower than those > 2.7 cm across in other *Rafflesia* species (Susatya 2020; Tolod et al. 2021). However, this approach cannot overestimate the time required for an unnoticeable bud to grow to a noticeable size. Based on this growth rate, the growth period for female (≤ 2.74 cm across) and male buds (≤ 2.72 cm across) was estimated to be 268 days (8.9 months) and 249 days (8.3 months), respectively.

Most of the observation on *Rafflesia* development and measurement started from a noticeable small round bud (cupule or post emergence stage) while still covered within the bark of its *Tetrastigma* host. The developmental period of this noticeable bud (ca. 2 cm across) to flowering stage varies between *Rafflesia* species, with 9–13 months in *R. tengku-adlinii*, 10–15 months in *R. pricei*, 12–16 months in *R. keithii* (Nais 2001) and 7.5 months in *R. patma* (Hidayati et al. 2000). The development of *R. arnoldii* from noticeable swellings (cupule stage) measured as small as 0.58–3.03 cm to flower took 11.6–16.9 months (Meijer 1958; Susatya 2020) while the 0.69–3.85 cm round swellings of *R. consueloae* took 12–14 months to flower.

Based on the actual bud development data, a female *R. cantleyi* bud of 2.75 cm in diameter took 313 days (10.4 months) to bloom, while the two male buds at 2.73 and 2.82 cm, took 287 (9.6 months) and 291 days (9.7 months), respectively. Therefore, combining these data of post-emergent stage with those in emergent stage, we estimated that female and male *R. cantleyi* buds would take 581 days (19.3 months) and 540–545 days (18.0–18.2 months) to develop from an unnoticeable emergent to flowering, respectively.

The observed blooming duration for *R. cantleyi* appeared similar to other *Rafflesia* species, i.e. between five and seven days, and depending on weather conditions. A few small fungus spots usually grow on the perigone lobes on the second or third day of a bloomed flower, depending on weather conditions (Refaei et al. 2011). The fungal colony would then spread to larger areas and cover all perigone lobes on the fifth to seventh day.

Fruits may take four to eight months to mature and ripen depending on species (Beaman and Adam 1984; Meijer 1997; Tolod et al. 2021; Whitten et al. 1996). Fruit herbivory and damage by animals may help seed dispersal and inoculation on the roots and stems of *Tetrastigma* hosts (Bänziger 1991; Bouman and Meijer 1994; Justesen 1922; Meijer 1958; Nais 2001; Pelser et al. 2013; Tolod et al. 2021). In this study, ants were seen visiting the fruit damaged by animals. Therefore, ants may be potentially an agent for seed dispersal and inoculation, as they may have contacted the horizontal vines as they move around the forest floor. The time estimated for successful seed inoculation and germination to an initial swelling on *Tetrastigma* hosts is between two to three years (Hidayati et al. 2000; Meijer 1997).

Based on the field data obtained in this study and published information on fruit formation and maturation, we estimated that the whole generative (visible) stage of *R. cantleyi* takes approximately 23.6–27.7 months (2.1–2.3 years). Extrapolating published information on the vegetative (invisible) stage, including seed dispersal, inoculation, germination and endophyte growth within the *Tetrastigma* host vines, which takes about 2–3 years (Hidayati et al. 2000; Meijer 1997), we estimated the duration of the life cycle for *R. cantleyi* spans about 4.0–5.3 years from seed to seed (Fig. 9). The estimated duration of *R. cantleyi*’s life cycle is closer to that of *R. arnoldii* (3.5–5 years), which was monitored from as small as 0.58 cm diameter buds by Susatya (2020) but longer than the published life cycle for most *Rafflesia* species, that is, between 3.0 and 4.5 years (Hidayati et al. 2000; Meijer 1997).

### Limitations and future studies

Despite being one of the very few long-term monitoring of *Rafflesia* populations, many questions still need to be answered to better elucidate the life history of *R. cantleyi*, particularly the influences of abiotic factors on the floral bud development and flowering success. The lack of comprehensiveness in ecological observations and our inability to trace each bud to individual hosts also limit our ability to interpret the population structure of *R. cantleyi* better.

Future studies combining more comprehensive and continuous monitoring of bud growth data and imagery (by different bud growth stages) in relation to soil physicochemical characteristics (such as soil type, moisture, pH, temperature, distance from water bodies, and slope inclination) along with local rainfall data of different populations could shed more light on the ecological significance of abiotic factors on bud development and mortality rates, subsequently life cycle duration of the visible stage and flowering success of the parasitic flowers in their natural habitats. These comprehensive abiotic parameters may also help explain the differences in the duration taken to complete the visible stage of the same *Rafflesia* species from different populations and geographical locations. Understanding such data has important consequences on both *in-situ* conservation and *ex-situ* propagation of *Rafflesia*.

In addressing the challenges encountered in delineating a *Rafflesia* population due to the endoparasite's cryptic life history, future studies could employ least-damaging samplings of tissue from flower buds and DNA-based clone identification for assigning each *Rafflesia* flower to an individual clone (Barkman et al. 2017). This could further examine the host internal control hypothesis on floral bud load per host plant (Susatya et al. 2017) and shed more light on the male–female flower dominance within a population to elucidate reproductive biology and connectivity between *Rafflesia* populations for sustainable management and conservation of *Rafflesia* in their natural ecosystems.

Most ecological studies have focused on the parasitic flower, while less attention was given to its *Tetrastigma* host. Investigating the physiological changes of internal nitrogen levels with flowering, fruiting and nutritional status of *T. rafflesiae* in relation to the bud development, mortality, and flowering success of *Rafflesia* may also shed more light on the host-parasite interactions. This information will better understand bud mortality due to nutrient demand and may also partly explain the host immune response and parasites' growth strategy within the *Tetrastigma* host in the vegetative stage of the *Rafflesia* life cycle.

## Conclusions

Here, we documented several new observations and estimated the duration of the life cycle of *R. cantleyi*. We showed that the mortality rate of buds, ranging between 30 and 77%, differed among sites in Lata Jarum, Raub, with the highest mortality rate for young buds below 5 cm in diameter. Similar to other *Rafflesia* species, the growth rate of *R. cantleyi* followed an exponential pattern, with female buds growing slightly slower than male buds. Interestingly, bud growth profiles were found significantly clustered according to the site, regardless of the sex of the flowers. The blooming rate also varied greatly between sites (23–56%). These observations prompt speculation that the physical attributes of sites may be an important factor influencing the growth rate and fate of *Rafflesia* buds. The populations of *R. cantleyi* exhibit a non-seasonal flowering pattern due to the lack of consistency and synchronisation between flowering frequency and rainfall pattern. The long monitoring period covering two discrete studies conducted on *R. cantleyi* populations at the same site has led to the discovery of the first female-biased *Rafflesia* population. The life cycle of *R. cantleyi* from seed to seed is estimated to be 4.0–5.3 years. The information yielded for *R. cantleyi* contributes to furthering our understanding of the life history of an endoparasite and provides valuable inputs for future study and the conservation of other *Rafflesia* species in the region.

## Data Availability

The data that support the findings of this study are available from the corresponding author upon reasonable request.
