# Correction to: Bud development, flower phenology and life history of holoparasitic *Rafesia cantleyi*

**DOI:** 10.1007/s10265-025-01633-9

**Published:** 2025-04-12

**Authors:** Suk Ling Wee, Shwu Bing Tan, Sue Han Tan, Bernard Kok Bang Lee

**Affiliations:** 1 Department of Biological Sciences and Biotechnology, Faculty of Science and Technology, Universiti Kebangsaan Malaysia, 43600 Bangi, Selangor Darul Ehsan, Malaysia; 2 Department of Mathematical Sciences, Faculty of Science and Technology, Universiti Kebangsaan Malaysia, 43600 Bangi, Selangor, Malaysia

**Correction to: Journal of Plant Research (2024) 137:423–443**

10.1007/s10265-024-01522-7

The article “Bud development, flower phenology and life history of holoparasitic *Rafesia cantleyi*”, written by Suk Ling wee, Shwu Bing Tan, Sue Han Tan and Bernard Kok Bang Lee, was originally published under exclusive license to the Author(s) under exclusive licence to The Botanical Society of Japan. As a result of the subsequent decision to publish the article under the open access model, the article’s copyright notice was changed on 17 March 2025 to © The Author(s) 2025 and the article is now distributed under a Creative Commons Attribution 4.0 International License, which permits use, sharing, adaptation, distribution and reproduction in any medium or format, as long as you give appropriate credit to the original author(s) and the source, provide a link to the Creative Commons licence, and indicate if changes were made. The images or other third party material in this article are included in the article’s Creative Commons Licence, unless indicated otherwise in a credit line to the material. If material is not included in the article’s Creative Commons Licence and your intended use is not permitted by statutory regulation or exceeds the permitted use, you will need to obtain permission directly from the copyright holder. To view a copy of this licence, visit http://creativecommons.org/licenses/by/4.0.

The original article has been corrected.

